# Draft Genome Assembly of *Pseudomonas aeruginosa* Quality Control Reference Strain Boston 41501

**DOI:** 10.1128/genomeA.00960-14

**Published:** 2014-10-02

**Authors:** T. D. Minogue, H. E. Daligault, K. W. Davenport, S. M. Broomall, D. C. Bruce, P. S. Chain, S. R. Coyne, H. S. Gibbons, J. Jaissle, O. Chertkov, T. Freitas, C. N. Rosenzweig, Y. Xu, S. L. Johnson

**Affiliations:** a Diagnostic Systems Division (DSD), United States Army Medical Research Institute of Infectious Diseases (USAMRIID), Fort Detrick, Maryland, USA; b Los Alamos National Laboratory (LANL), Los Alamos, New Mexico, USA; c United States Army Edgewood Chemical Biological Center (ECBC), Aberdeen Proving Ground, Aberdeen, Maryland, USA

## Abstract

We present the scaffolded genome assembly of *Pseudomonas aeruginosa* Boston 41501, now publicly available in GenBank (JOVK00000000) in 10 contigs placed into a single scaffold. The 6.82-Mbp genome contains 66.1% G+C content and 6,295 coding sequences, including type 4 pilus and type 3 secretion system production genes.

## GENOME ANNOUNCEMENT

*Pseudomonas aeruginosa* is a Gram-negative member of the *Gammaproteobacteria* found in a wide variety of environments (soil water, animals, humans, and hospitals). *P. aeruginosa* Boston 41501 (ATCC 27853) is a quality control strain often used in opportunistic pathogen research and originally isolated from a blood culture. Most human infections are hospital acquired and/or limited to immunocompromised persons. Recently the U.S. Centers for Disease Control and Prevention elevated the risk of antibiotic-resistant *P. aeruginosa* infection to “serious” due to estimates of 6,700 cases and 400 deaths annually (1).

High-quality genomic DNA was extracted from a stationary-phase purified isolate using a Qiagen Genomic-tip 500 at the USARMIID Diagnostic Systems Division (DSD) per the manufacturer’s recommendations. Draft sequence data included a 100-bp Illumina library (128-fold genome coverage) and a separate long-insert paired-end library (8,792- ± 2,198-bp insert, 35-fold genome coverage) (Roche 454 Titanium platform). The two data sets were assembled together in Newbler (Roche) and the consensus sequences computationally shredded into 2-kbp overlapping fake reads (shreds). The raw reads were also assembled in Velvet and those consensus sequences computationally shredded into 1.5-kbp overlapping shreds (2). Draft data from all platforms were then assembled together with Allpaths and the consensus sequences computationally shredded into 10-kbp overlapping shreds (3). We then integrated the Newbler consensus shreds, Velvet consensus shreds, Allpaths consensus shreds, and a subset of the long-insert read pairs using parallel Phrap (High Performance Software, LLC). Possible misassemblies were corrected, and some gap closure was accomplished with manual editing in Consed (4–6).

Automatic annotation of the *P. aeruginosa* Boston 41501 genome utilized an Ergatis-based workflow at LANL with minor manual curation. The final annotated assembly (6,295 coding sequences, 31 rRNAs, and 66 tRNAs in the 6,819,384-bp genome sequence) is available in GenBank (accession number JOVK00000000), and raw data can be provided upon request. Preliminary review of the annotations finds at least 28% of the virulence genes noted by Feinbaum et al. (7) and both type 4 pilus and type 3 secretion systems (8).

### Nucleotide sequence accession number.

This genome sequence is available in GenBank under the accession number JOVK00000000.

## References

[1] Centers for Disease Control and Prevention (CDC) 2013 Antibiotic resistance threats in the United States. U.S. Centers for Disease Control and Prevention, Atlanta, GA. http://www.cdc.gov/drugresistance/threat-report-2013/pdf/ar-threats-2013-508.pdf

[2] Zerbino DR Birney E 2008 Velvet: algorithms for *de novo* short read assembly using de Bruijn graphs. Genome Res. 18:821–829. 10.1101/gr.074492.107 18349386 PMC2336801

[3] Butler J MacCallum I Kleber M Shlyakhter IA Belmonte MK Lander ES Nusbaum C Jaffe DB 2008 ALLPATHS: *de novo* assembly of whole-genome shotgun microreads. Genome Res. 18:810–820. 10.1101/gr.7337908 18340039 PMC2336810

[4] Ewing B Hillier L Wendl MC Green P 1998 Base-calling of automated sequencer traces using phred. I. Accuracy assessment. Genome Res. 8:175–185. 10.1101/gr.8.3.175 9521921

[5] Ewing B Green P 1998 Base-calling of automated sequencer traces using phred. II. Error probabilities. Genome Res. 8:186–194 9521922

[6] Gordon D Abajian C Green P 1998 Consed: a graphical tool for sequence finishing. Genome Res. 8:195–202. 10.1101/gr.8.3.195 9521923

[7] Feinbaum RL Urbach JM Liberati NT Djonovic S Adonizio A Carvunis AR Ausubel FM 2012 Genome-wide identification of *Pseudomonas aeruginosa* virulence-related genes using a *Caenorhabditis elegans* infection model. PLOS Pathog. 8 e1002813. 10.1371/journal.ppat.1002813 22911607 PMC3406104

[8] Gellatly SL Hancock RE 2013 *Pseudomonas aeruginosa*: new insights into pathogenesis and host defenses. Pathog. Dis. 67:159–173. 10.1111/2049-632X.12033 23620179

